# The cost and impact of male circumcision on HIV/AIDS in Botswana

**DOI:** 10.1186/1758-2652-12-7

**Published:** 2009-05-27

**Authors:** Lori A Bollinger, John Stover, Godfrey Musuka, Boga Fidzani, Themba Moeti, Lesego Busang

**Affiliations:** 1 Futures Institute, Glastonbury, Connecticut, USA; 2 African Comprehensive HIV/AIDS Partnership, Gaborone, Botswana; 3 National AIDS Coordinating Agency, Gaborone, Botswana

## Abstract

The HIV/AIDS epidemic continues to be a major issue facing Botswana, with overall adult HIV prevalence estimated to be 25.7 percent in 2007. This paper estimates the cost and impact of the draft Ministry of Health male circumcision strategy using the UNAIDS/WHO Decision-Makers' Programme Planning Tool (DMPPT). Demographic data and HIV prevalence estimates from the recent National AIDS Coordinating Agency estimations are used as input to the DMPPT to estimate the impact of scaling-up male circumcision on the HIV/AIDS epidemic. These data are supplemented by programmatic information from the draft Botswana National Strategy for Safe Male Circumcision, including information on unit cost and program goals. Alternative scenarios were developed in consultation with stakeholders. Results suggest that scaling-up adult and neonatal circumcision to reach 80% coverage by 2012 would result in averting almost 70,000 new HIV infections through 2025, at a total net cost of US$47 million across that same period. This results in an average cost per HIV infection averted of US$689. Changing the target year to 2015 and the scale-up pattern to a linear pattern results in a more evenly-distributed number of MCs required, and averts approximately 60,000 new HIV infections through 2025. Other scenarios explored include the effect of risk compensation and the impact of increasing coverage of general prevention interventions. Scaling-up safe male circumcision has the potential to reduce the impact of HIV/AIDS in Botswana significantly; program design elements such as feasible patterns of scale-up and inclusion of counselling are important in evaluating the overall success of the program.

## Background

The HIV/AIDS epidemic continues to be a major issue facing Botswana, with overall adult HIV prevalence estimated to be 25.7% in 2007 [1]. As an add-on strategy to augment its efforts to reduce HIV prevalence, the Ministry of Health has drafted a male circumcision strategy [2]. In addition, there has been a significant increase in the provision of antiretroviral therapy (ART) in Botswana, which also has an impact on HIV prevalence levels.

Male circumcision has been shown to reduce HIV transmission from females to males in various settings. Three randomized controlled trials, in South Africa, Uganda and Kenya, showed that HIV transmission from females to males was reduced by up to 60% when male circumcision was undertaken [3-5]. In countries where the prevalence of male circumcision is low, as in Botswana, there is great potential to reduce HIV prevalence rates through implementing interventions offering safe male circumcision [6].

Several modelling studies have been published recently for various countries in sub-Saharan Africa that examine the impact of scaling up male circumcision on HIV incidence and prevalence levels, including Kenya [7], Uganda [8], South Africa [9,10], and southern Africa in general [11,12]. The reduction in HIV incidence rates after 10 years in these studies varies from 10% to 55%, while decreases in HIV prevalence rates after 10 years varies from 17% to 50%.

Because of this high level of effectiveness, Botswana is exploring the future costs and impact of implementing safe male circumcision. The purpose of this research is to estimate the overall cost and impact of a scaled-up programme of safe male circumcision in Botswana, including the impact of alternative scenarios.

## Methods

Demographic estimates and projections of impact of HIV prevalence use data from the recent estimates and simulations undertaken by the Botswana National AIDS Coordinating Agency [13] as input to the male circumcision Decision-Makers' Programme Planning Tool (DMPPT) [14] of the Joint United Nations Programme on HIV/AIDS (UNAIDS) and World Health Organization (WHO). The epidemiologic research utilized antenatal clinic sentinel surveillance data and the Botswana AIDS Impact Survey of 2004 to estimate HIV prevalence in Botswana from 1980 to 2007 using the UNAIDS Epidemic Projection Package [15].

The national-level prevalence projection was then combined with age-specific and sex-specific HIV prevalence data from the 2004 impact survey in the AIM module of Spectrum [16] to calculate the number of people infected with HIV, including new infant infections based on the programmes in Botswana that currently provide antiretroviral prophylaxis and replacement feeding, as well as other relevant indicators.

These estimates are then used in the UNAIDS/WHO DMPPT, which calculates the cost of male circumcision services by delivery mode, based on clinical guidelines and local costs for both direct and shared facility and staff costs. The tool then estimates the impact of the epidemic using a transmission model that calculates new infections by age and sex as a function of the current force of infection, coverage levels, and speed of scale up.

This model is intended to support policy development and planning for scaling up services to provide male circumcision. It allows analysts and decision makers to understand the costs and impacts of policy options, and is a part of a larger tool kit developed by UNAIDS/WHO that provides guidelines on comprehensive approaches to male circumcision, including types of surgical procedures and key policy and cultural issues.

The data from the National AIDS Coordinating Agency estimates are supplemented by programmatic information from the draft national male circumcision strategy, including information on unit cost and programme goals. In addition, a stakeholders' workshop was held in Botswana's capital city, Gaborone, on 2 October 2008, during which data assumptions were reviewed and scenarios developed.

The male circumcision (MC) model has two components: costing and impact. The initial unit cost of an uncomplicated adult male circumcision of US$48 in the public sector was provided in the national strategy, so the costing component of the model was not applied in Botswana.

Three other unit costs were developed and agreed upon during the workshop: a neonatal circumcision unit cost of $38 (assumed to be 20% lower than the adult cost, due to lower complication rates and lower costs for commodities), and private provider unit costs for both adult and neonatal circumcisions of $60 and $48 (assumed to be 25% higher than the relevant public sector costs). In addition, user fees of $1 for public sector and $25 for private providers were assumed.

The impact component of the model addresses the following key policy areas:

• priority populations: a choice of all male adults, young adults, adolescents, newborns, and men at higher risk of HIV exposure

• target coverage levels and rates of scale up

• service delivery modes: hospital, clinic, mobile van; public, private, non-governmental organization; and "other"

• impact of other prevention activities and risk compensation effects.

A wide variety of inputs is required, including:

• demographic: size of various population groups

• epidemiological: HIV prevalence rates for overall population and specific groups, underlying transmission factors (based on scientific evidence), effectiveness of male circumcision in reducing transmission

• sexual behaviour: sexual mixing matrix

• programmatic: target populations, rate and timing of scale up, service delivery mode

• economic: unit cost (described above), discounted lifetime cost of ART.

A complete listing of the data and assumptions used in the model will be provided upon request. Much of the data are derived from either the Botswana AIDS Impact Survey II or the national agency estimates.

The programmatic decisions, such as rate and timing of scale up, were agreed upon during the stakeholders' workshop. Some of the parameters were varied in order to perform sensitivity analyses; the specific scenarios presented here were discussed during the stakeholders' workshop.

## Results and discussion

### Scenario 1: national strategy scenario

Scenario 1 presents the impact of increasing the prevalence of male circumcision to 80% of HIV-negative adult males and neonates by 2012, based on the objectives expressed in the national strategy. It is assumed that the scaling up begins in 2009 and follows an S-shaped pattern to allow for training of physicians and other infrastructure developments. Although the scaling up is completed by 2012, results are presented through 2025 in order to measure the long-term impact of increasing the prevalence of male circumcision.

**Figure 1:**
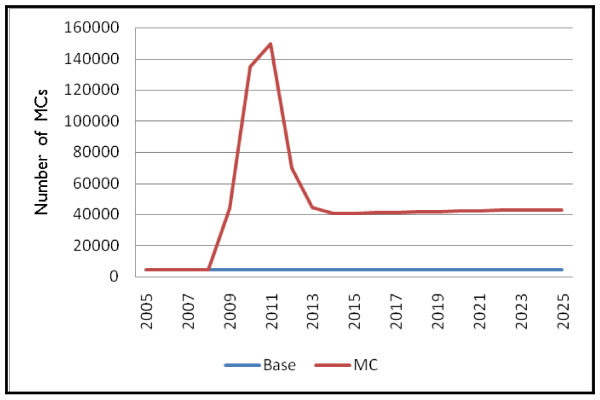
**Number of male circumcisions performed by scenario**.

Figure 1 presents the number of male circumcisions performed for the "Base" scenario, where the current prevalence rate of MC is held constant at the initial level of 10.2% throughout the time period, and the "MC" scenario, where circumcision is scaled up according to the pattern described for Scenario 1.

The number of circumcisions performed in the "Base" scenario reflects the constant MC prevalence rate specified over the time period remaining relatively constant at around 4700 per year.

When safe male circumcision is scaled up to reach a prevalence rate of 80% by 2012, there is a rapid increase in the number of circumcisions performed for the first four years of the scenario, as the programme plays catch up with the stock of uncircumcised men, reaching a peak of over 140,000. By 2013, the number begins to drop, and the final number required levels off to reach a rate of about 43,000 circumcisions per year for the duration of the time period.

**Figure 2:**
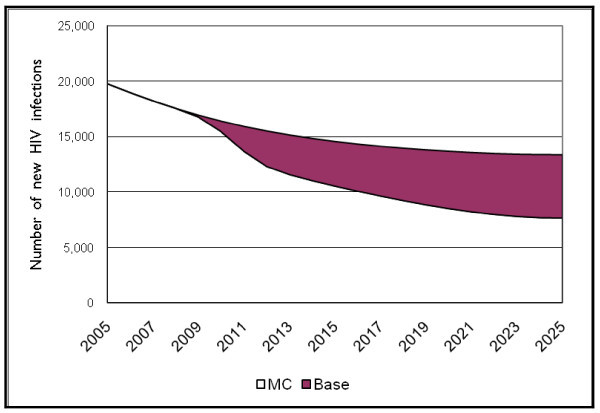
**New adult HIV infections by scenario**.

There is a strong impact of scaling up safe male circumcision on the number of new adult infections (see Figure 2). While the number of new infections declines in the "Base" scenario from about 18,000 in 2007 to 13,400 by 2025, the number of new infections in the MC scenario declines even further, to reach about 7600 by 2025. Note that the decline starts when the programme begins to scale up, but then continues throughout the time period, illustrating why it is important to show the impact of the MC for a longer time period.

Overall, between 2008 and 2015, about 18,000 cumulative HIV infections, or 14% of total new HIV infections, are averted. Over the next 10-year time period (2016–2025), the number of cumulative new HIV infections averted reaches more than 51,000, or 38% of all new infections.

**Figure 3:**
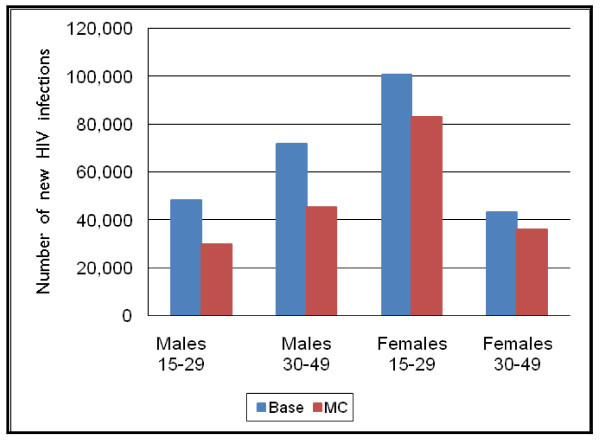
**New adult HIV infections by scenario by age and sex, 2008–2025**.

Although the primary impact of increasing the prevalence of male circumcision is to reduce the number of new HIV infections in men, the number of new HIV infections in women is also reduced via secondary impacts. Figure 3 shows the cumulative impact from 2008–2025 of increasing male circumcision on both males and females, split into the two different age groups (15 to 29, and 30 to 49).

The cumulative number of new HIV infections for men drops by over 18,000 for those aged 15 to 29 and by about 26,000 for those aged 30 to 49. The cumulative number of new HIV infections for women drops by more than 17,000 for those aged 15 to 29 and by about 7000 for those aged 30 to 49.

The number of male circumcisions that are required in order to avert one HIV infection is calculated by dividing the increase in the number of male circumcisions performed by the number of HIV infections averted over the relevant time period:

where X = number of male circumcisions

Y = number of HIV infections, and

t = 2008–2015 and 2016–2025.

Between 2008 and 2015, 27.3 male circumcisions are required in order to avert one HIV infection. Because of the increasing impact of circumcision over time, however, this statistic decreases when it is calculated for the time period 2016–2025, reaching a low value of 7.3 circumcisions that need to be performed in order for an infection to be averted.

Note that this is an upper bound for this statistic as the impact of including male circumcision (especially neonatal MC) will extend beyond the current time horizon of the model. These statistics can be compared to similar statistics from other studies, including a figure of six for Lesotho, four for Swaziland, and eight for Zambia [17].

The final piece to the puzzle is the cost of the programme, which needs to be evaluated relative to its effectiveness. Note that, in addition to the unit costs of male circumcision discussed here, the national strategy calls for spending 14.4 million Botswana pula (P), which equals about US$2.3 million, over five years to generate demand. Also note that after discussion, experts agreed that approximately 80% of male circumcisions would take place in the public sector, and approximately 20% would be performed by private providers.

**Figure 4:**
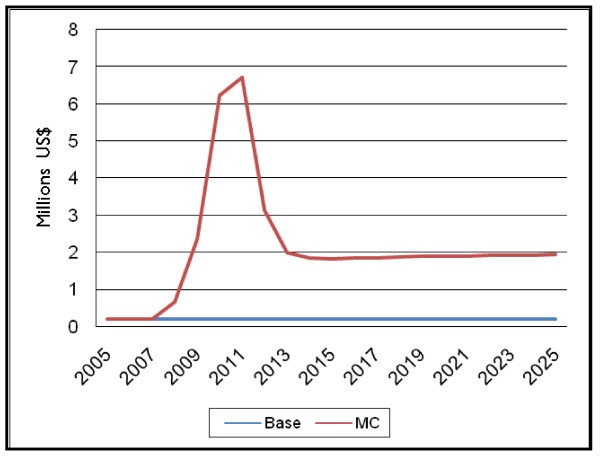
**Total net cost of male circumcision programme in US dollars (net of user fees collected)**.

The total net cost of the new male circumcision programme reaches a peak of $6.5 million, and then returns to a stable level of about $1.7 million per year required to maintain a circumcision prevalence rate of 80%, where net cost is defined as the total cost of all male circumcisions performed in all service delivery modes, less any user fees collected. In contrast, in the "Base" scenario, the current expenditure on male circumcision remains at about $200,000 for the duration of the timeframe (see Figure 4).

The total cost by year is displayed in the first two columns of Table 1, along with the incremental cost in the final column, and the cumulative total for two time periods in the last two rows. The cumulative cost for implementing a scaled-up MC programme through 2015 is $23 million, while the cumulative cost between 2016 and 2025 is about $17 million, resulting in a total cumulative cost for scaled-up male circumcision through 2025 of approximately $40 million.

**Table 1:** Net cost of scaled-up male circumcision programme (net of user fees collected)

Year	Base projection	MC projection	Additional cost
2005	207,345	207,345	0
**2006**	**207,395**	**207,395**	**0**
2007	207,445	207,445	0
**2008**	**207,495**	**665,114**	**457,619**
2009	207,545	2,346,725	2,139,180
**2010**	**207,596**	**6,224,314**	**6,016,718**
2011	207,646	6,706,579	6,498,933
**2012**	**207,696**	**3,123,637**	**2,915,941**
2013	207,746	2,005,039	1,797,292
**2014**	**207,797**	**1,842,539**	**1,634,743**
2015	207,847	1,833,937	1,626,090
**2016**	**207,897**	**1,846,128**	**1,638,231**
2017	207,947	1,860,367	1,652,420
**2018**	**207,998**	**1,874,046**	**1,666,048**
2019	208,048	1,886,770	1,678,722
**2020**	**208,098**	**1,898,452**	**1,690,354**
2021	208,149	1,909,053	1,700,904
**2022**	**208,199**	**1,918,568**	**1,710,369**
2023	208,249	1,927,124	1,718,875
**2024**	**208,300**	**1,934,720**	**1,726,420**
2025	208,350	1,938,336	1,729,986
**2008–2015**	**1,661,368**	**24,747,884**	**23,086,516**
2016–2025	2,081,235	18,993,563	16,912,328

Combining this result with the number of HIV infections averted results in a calculation of discounted net cost per HIV infection averted. Overall, the discounted net cost per HIV infection averted for the time period 2008–2015 is $1353. When the discounted net costs and number of infections averted are evaluated for the entire time period of the scenario, 2008–2025, the discounted net cost per HIV infection averted drops to $642.

In addition, net savings per HIV infection averted are calculated as the savings due to future ART costs avoided, minus the net circumcision costs, where the discounted lifetime cost of ART is based on a unit cost of P3599 in 2010 and P4135 in 2015 for first-line antiretrovirals (ARVs) with an additional $133 for second-line ARVs [18].

In addition, we assume continuation rates on ART of 91% for the first year and 99% for subsequent years [16]. The net savings, assuming a discounted lifetime cost of ART of $11,258, equals $9905 for the time period 2008–2015, and $10,616 when evaluated across the entire time period.

Net costs and savings can also be calculated relative to the number of male circumcisions performed. Net costs relative to MC performed remains about the same across the two time periods, while the net savings per circumcision performed increases substantially once the savings are evaluated over the entire time period, from $200 to $427.

Finally, a sensitivity analysis can be performed for some of the key parameters, including: the reduction in female-to-male transmission, which is assumed initially to be 60%; the reduction in male-to-female transmission, which is assumed initially to be 0%; the discount rate, which is assumed initially to be 3%; and the discounted lifetime cost of ART, which is assumed initially to be $11,000 for the purposes of this sensitivity analysis. The results of the sensitivity analysis are shown in Table 2. Each of the initial values of the parameters in the sensitivity analysis is shown in bolded, italicized font.

**Table 2:** Sensitivity analysis of key parameters (2008–2025)

	Parameter values	Infections averted	Number of circumcisions per infection averted	Cost net of user fees per infection averted	Cost savings per infection averted
Base case		51,518	7.3	$642	$10,616
Effectiveness	30%	25,059	14.7	$1,313	$9,945
	***60%***	***51,518***	***7.3***	***$642***	***$10,616***
	75%	65,218	5.8	$508	$10,750
Reduction in M->F transmission	***0%***	***51,518***	***7.3***	***$642***	***$10,616***
	30%	67,444	5.7	$473	$10,785
Discount rate	***3%***	***51,518***	***7.3***	***$642***	***$10,616***
	5%	51,518	7.3	$691	$10,567
	7%	51,518	7.3	$743	$10,515
Lifetime cost of ART	$8,000	51,518	7.3	$642	$7,358
	***$11,000***	***51,518***	***7.3***	***$642***	***$10,358***
	$14,000	51,518	7.3	$642	$13,358
	Minimum	25,059	5.7	$473	$7,358
	***Base Case***	***51,518***	***7.3***	***$642***	***$10,616***
	Maximum	67,444	14.7	$1,313	$13,358

The results are as expected, and help to confirm the robustness of the model. If the effectiveness of male circumcision on the transmission probability is reduced so that the transmission rate is relatively higher, the number of HIV infections averted decreases, and the cost per infection averted increases. If instead the effectiveness of circumcision is higher, so that the transmission probability is reduced even further than the initial reduction of 60%, then the number of infections averted increases, and the net cost per infection decreases.

If the cost net of user fees of circumcision increases because of a higher discount rate, then the cost per infection averted increases. If the discounted lifetime cost of ART increases (decreases), then the effect is to increase (decrease) the net savings per HIV infection averted.

Given these results, the stakeholders' workshop recommended exploring further scenarios:

• **Scenario 2**: What is the impact of changing the target date of full coverage from 2012 to 2015?

• **Scenario 3**: What is the impact of reversing behaviour change that may occur due to risk compensation effects?

• **Scenario 4**: What is the impact of increasing the coverage of other general prevention programmes to 80% with a resulting decline in risky behaviours of 35%?

### Scenario 2: target date of 2015

Because of the huge number of male circumcisions that would be required in order to reach the specified target of 80% by 2012, an alternative scenario was suggested during the workshop where the target date is changed to 2015, and the scale-up pattern is linear rather than S-shaped.

**Figure 5:**
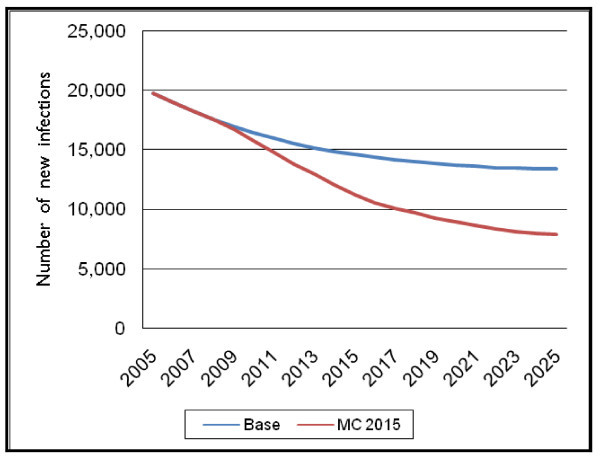
**Number of new adult HIV infections for Scenario 2 – 2015 target year**.

These changes result in smoothing out the number of total male circumcisions required over the time period, with a peak of around 85,000 circumcisions required in 2015 compared to the peak of almost 150,000 circumcisions required in Scenario 1. The impact on the number of new adult HIV infections of postponing the target year to 2015 can be seen in Figure 5.

Here, the number of new adult HIV infections declines as well, reaching a level of 7900 by 2025, which is somewhat higher than the 7673 reached by 2025 in the initial scenario. Postponing the target year to 2015 and changing the scale-up pattern to linear averts approximately 60,000 new adult HIV infections cumulatively through 2025, while the initial scenario resulted in approximately 70,000 new adult HIV infections averted through 2025.

Because of the lower number of infections averted, the net cost per infection averted increases slightly, from $1353 to $1953 over the time period 2008–2015, and from $642 to $759 over the time period 2008–2025.

### Scenario 3: risk compensation

Another concern that was raised during the workshop was whether, despite the counselling that would take place, men circumcised through this intervention and their partners would begin practicing riskier sex due to perceived risk reduction as a result of circumcision. Although the biological impact of male circumcision is to reduce HIV transmission, this impact might be ameliorated if behaviour reversals occur.

The model calculates the impact of risky sexual behaviours reverting to patterns that existed earlier in the epidemic, prior to the roll-out of the MC programme. Note that this impact is not due to early resumption of sexual activity, but instead is the result of changing back to previous sexual behaviours, such as lower condom use and/or more sexual partners.

The "Base" case in this instance is Scenario 1, the national strategy scenario; "MC" is the previous scenario; and "RC" is the result if risk compensation (RC) occurs (in this case, if 50% of circumcised men reverse their behaviour to previous, riskier sexual behaviour).

**Figure 6:**
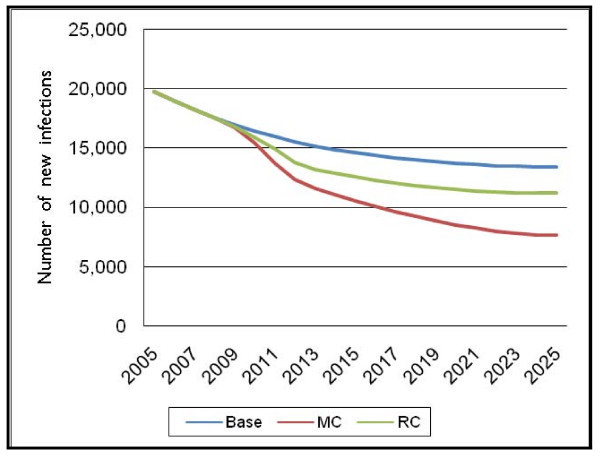
**Number of new adult HIV infections for Scenario 3 – risk compensation effects**.

If half of newly circumcised men revert to sexual behaviours they practiced before being circumcised, the number of new HIV infections decreases relative to the "Base" case, but at a lower rate than before, as shown in the "RC" scenario (see Figure 6). Thus it is imperative that appropriate counselling occurs during the visits prior to and following the actual male circumcision procedure.

### Scenario 4: increasing coverage of general prevention interventions

The last scenario to be explored is the impact of increasing the coverage of general prevention interventions from 20% to 80%, along with increasing the male circumcision prevalence rate. The impact is calculated as a proportional reduction in the force of infection at full coverage due to behaviour changes, such as increasing condom use and decreasing number of partners.

**Figure 7:**
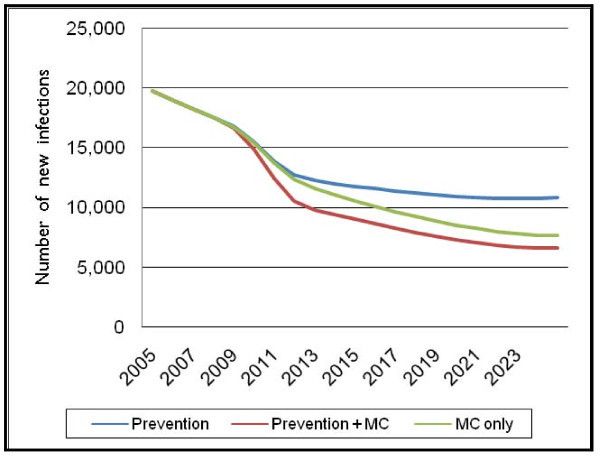
**Number of new adult HIV infections for Scenario 4 – full prevention coverage**.

Although the default value of the proportional reduction is 70%, the consensus at the workshop was that this proportional reduction should be half the default value, or a 35% reduction in the force of infection, in Botswana, due to country-specific characteristics (see Figure 7).

The initial impact of increasing the coverage of general prevention interventions can be seen in the "Prevention" case, where the number of new adult HIV infections reaches a new value of approximately 10,800 in 2025, compared to the previous value of 13,400 in 2025 in the first scenario.

Adding the impact of a scaled-up MC programme to the scaled-up prevention results in a further decline in the number of new adult HIV infections – to a level of 6600 in 2025 in the "Prevention+MC" scenario. This is even lower than the level of 7700 in the scenario "MC only", which is the result of the initial scenario. Thus the level of new adult HIV infections is lower once male circumcision programmes are scaled up, even if other, more general prevention programmes are scaled up as well.

## Conclusion

As part of its long-term planning process, the Botswana Ministry of Health and the National AIDS Coordinating Agency requested analyses regarding the cost and impact of scaling up safe male circumcision. Building on previous HIV prevalence estimates recently completed in Botswana, the UNAIDS/WHO DMPPT for male circumcision was applied in Botswana, including participation of various stakeholders in a workshop on 2 October 2008 to validate the data inputs and policy assumptions used in the MC model.

Results from the MC model suggest that scaling up adult and neonatal circumcision to reach 80% coverage by 2012 would result in averting almost 70,000 new HIV infections through 2025, at a total net cost of $47 million across that same period, resulting in an average cost per HIV infection averted of $642.

Although scaling up coverage to 80% by 2012 using an S-shaped pattern would have a significant impact on reducing the number of new HIV infections, it would also require circumcising a huge number of men; male circumcisions required reach almost 150,000 in 2011 before levelling off to about 43,000. Changing the target year to 2015 and the pattern to a linear pattern results in a more evenly distributed number of circumcisions required, and averts approximately 60,000 new HIV infections through 2025.

Concerns were raised at the stakeholders' workshop that sexual behaviour may change as a result of scaling up male circumcisions in Botswana. Risk compensation effects could have a significant impact on reversing the gains that increasing safe circumcisions would have on the HIV/AIDS epidemic in Botswana; modelling results imply that if 50% of all newly circumcised men returned to the previous level of risky behaviours, the positive impact of safe circumcisions would be cut in half.

Finally, in response to the current policy environment in Botswana where other prevention interventions are also being scaled up, the impact of increasing this coverage was evaluated on its own, and in addition to scaling up circumcisions. Results show that scaling up other prevention interventions would result in a lower level of HIV prevalence than before, but there would still be a significant impact if safe male circumcisions were added to the general package of other prevention interventions.

## Competing interests

The authors declare that they have no competing interests.

## Authors' contributions

LAB participated in the design of the study, performed the statistical analysis and drafted the manuscript. JS participated in the design of the study and the statistical analysis. BF, GM, TM and LB participated in the design and coordination of the study. All authors read and approved the final manuscript.

## References

[B1] National AIDS Coordinating Agency HIV/AIDS in Botswana: Estimated Trends and Implications Based on Surveillance and Modeling. Gaborone 2008

[B2] Ministry of Health, Republic of Botswana Botswana Safe Male Circumcision Add-on Strategy for HIV Prevention, DRAFT. Gaborone 2008

[B3] Auvert B Taljaard D Lagaarde E Sobngwi-Tambekou J Sitta R Puren A Randomized, controlled intervention of male circumcision for reduction of HIV infection risk: the ANRS 1265 trial PLoS Med 2005 12 11 e298 10.1371/journal.pmed.0020298 16231970 PMC1262556

[B4] Gray RH Kigozi G Serwadda D Makumbi F Watya S Nalugoda F Kiwanuka N Moulton LH Chaudhary MA Chen MZ Sewankambo NK Wabwire-Mangen F Bacon MC Williams CF Opendi P Reynolds SJ Laeyendecker O Quinn TC Wawer MJ Male circumcision for HIV prevention in men in Rakai, Uganda: a randomized, controlled trial Lancet 2007 12 9562 657 66 10.1016/S0140-6736(07)60313-4 17321311

[B5] Bailey RC Moses S Parker CB Agot K Maclean I Krieger JN Williams CF Campbell RT Ndinya-Achola JO Male circumcision for HIV prevention in young men in Kisumu, Kenya: a randomized, controlled trial Lancet 2007 12 9562 643 56 10.1016/S0140-6736(07)60312-2 17321310

[B6] Williams BG Lloyd-Smith JO Gouws E Hankins C Getz WM Hargrove J de Zoysa I Dye C Auvert B The potential impact of male circumcision on HIV in Sub-Saharan Africa PLoS Med 2006 12 7 e262 10.1371/journal.pmed.0030262 16822094 PMC1489185

[B7] Nagelkerke NJ Moses S de Vlas SJ Bailey RC Modelling the public health impact of male circumcision for HIV prevention in high prevalence areas in Africa BMC Infect Dis 2007 12 16 10.1186/1471-2334-7-16 17355625 PMC1832203

[B8] Gray RH Li X Kigozi G Serwadda D Nalugoda F Watya S Reynolds SJ Wawer M The impact of male circumcision on HIV incidence and cost per infection prevented: a stochastic simulation model from Rakai, Uganda AIDS 2007 12 845 850 10.1097/QAD.0b013e3280187544 17415039

[B9] Kahn JG Marseille E Auvert B Cost-effectiveness of male circumcision for HIV prevention in a South African setting PLoS Med 2006 12 e517 10.1371/journal.pmed.0030517 17194197 PMC1716193

[B10] Auvert B Marseille E Korenromp EL Lloyd-Smith J Sitta R Taljaard D Pretorius C Williams B Kahn JG Estimating the resources needed and savings anticipated from roll-out of adult male circumcision in Sub-Saharan Africa PLoS ONE 2008 12 e2679 10.1371/journal.pone.0002679 18682725 PMC2475667

[B11] Londish GJ Murray JM Significant reduction in HIV prevalence according to male circumcision intervention in sub-Saharan Africa Int J Epidemiol 2008 12 1246 1253 10.1093/ije/dyn038 18316348

[B12] Podder CN Sharomi O Gumel AB Moses S To cut or not to cut: a modeling approach for assessing the role of male circumcision in HIV control Bull Math Biol 2007 12 2447 2466 10.1007/s11538-007-9226-9 17557187

[B13] Stover J Fidzani B Molomo BC Moeti T Musuka G Estimated HIV trends and program effects in Botswana PLoS ONE 2008 12 11 e3729 doi:10.1371/journal.pone.0003729 10.1371/journal.pone.0003729 19008957 PMC2579326

[B14] UNAIDS Decision-Makers' Programme Planning Tool for Male Circumcision Scale-up. Model and manual http://futuresinstitute.org/pages/resources.aspx

[B15] Brown T Grassly NC Garnett G Stanecki K Improving projections at the country level: the UNAIDS Estimation and Projection Package 2005 Sex Trans Inf Sex Transm Infect 2006 12 Suppl 3 34 40 10.1136/sti.2006.020230 PMC2576727 16735291

[B16] Stover J AIM: A Computer Program for Making HIV/AIDS Projections and Examining the Demographic and Social Impacts of AIDS 2009 Washington, DC: USAID | Health Policy Initiative

[B17] Martin G Bollinger L Pandit-Rajani T Tshelo R Nkambula R Forsythe S Stover J Costing Male Circumcision in Lesotho, Swaziland and Zambia: Implications for the Cost-Effectiveness of Male Circumcision as a Cost-Effective Intervention for HIV Prevention 2007 Washington DC; USAID Health Policy Initiative

[B18] ACHAP Roadmap to Sustainability Report. Gaborone 2008

